# Die katholische Welt und die Urologie im 20./21. Jahrhundert

**DOI:** 10.1007/s00120-023-02152-y

**Published:** 2023-09-06

**Authors:** Florian G. Mildenberger

**Affiliations:** Institut für Geschichte, Theorie und Ethik der Medizin, Heinrich Heine Universität Düsseldorf, Moorenstr. 5, 40225 Düsseldorf, Deutschland

**Keywords:** Glaubenslehre, Verhütung, Ehe, Impfung, Naturheilkunde, Masturbation, Ethik, Doctrine of faith, Contraception, Marriage, Vaccination, Naturopathy, Masturbation, Ethics

## Abstract

Die katholische Welt ist geprägt durch päpstliche Vorgaben und ihre Interpretation im Wandel der Zeit durch berufene Theologen. Es bestehen strenge Verbote bei zentralen Fragestellungen über Leben und Tod, aber im Rahmen der praktischen Anwendung ärztlicher Behandlungstechniken bleibt der Vatikan häufig vage. Dies kann katholischen Urologen erlauben, eine Reihe von Therapien anzuwenden, die auf den ersten Blick problematisch erscheinen. Bedienen sich die Ärzte zudem einer anderen Ausdrucksweise als derjenigen, die in der Glaubenslehre genannt wird, besteht ein größerer Handlungsspielraum.

## Abstract (en)

The Catholic world is shaped by papal directives and their interpretation over time by appointed theologians. There are strict prohibitions on key questions about life and death, but in the context of the practical application of medical treatment techniques, the Vatican often remains vague. This may allow Catholic urologists to use a range of therapies that at first glance appear problematic. Furthermore, if physicians use a different form of expression than that stated in the doctrine of the faith, there is more space for medical practice.

## Zur Einleitung – selbst verschuldete Probleme

Die katholische Kirche ist weiterhin eine geistige Weltmacht ohne formale weltliche Autorität. Gleichwohl gelten die Verlautbarungen, Lehrsätze und Erklärungen des Vatikans für viele Menschen auf dem Globus weit bindender als weltliche Gesetze in den jeweiligen Staaten. Sie sind Ausdruck einer normativen Ethik, der jede individuelle Betrachtung akuter Probleme fehlt. Dies führt, gerade im medizinisch-ethischen Bereich, häufig zu Konflikten.

Der Vatikan als Denkfabrik und Ordnungszentrale der katholischen Welt mischt sich zugleich nicht in alle Teilbereiche des Lebens ein. Es werden übergeordnete Anordnungen verfasst, die aber nicht ins Detail gehen. So kommt beispielsweise die Urologie als ärztliche Disziplin in keiner Enzyklika vor. Eine *Enzyklika* ist ein Rundschreiben an alle Gläubigen des Erdkreises und für diese bindend. Ein *Lehrschreiben* hingegen richtet sich an eine spezielle Gruppe von Gläubigen und kann – wenn es sich beispielsweise direkt an Bischöfe wendet – durch diese weiter verbreitet werden. Darüber hinaus gibt es *Hirtenbriefe*, die sich explizit nur an Mitglieder der Kirche in einer bestimmten Region richten.

Päpstliche Verlautbarungen über Beginn und Ende des Lebens, die ethische Rechtmäßigkeit bestimmter ärztlicher Handlungen und göttliche Eingriffe in die bestehende Welt, gegen die nicht vorzugehen sei, spielen auch in der urologischen Praxis eine Rolle.

Die sich rasant beschleunigende Entwicklung der medizinisch-technischen Geräte, Theorien und Therapien überfordert jedoch spätestens seit dem Beginn des 20. Jahrhunderts den Rezeptionshorizont katholischer Theologen, weshalb päpstliche Verlautbarungen und Regelwerke bisweilen mit Jahren oder Jahrzehnten Verspätung erlassen werden. Diese Form des theologisch-ethischen Interregnums nutzten in der Vergangenheit wirkmächtige Moraltheologen, um ihre Sicht der Dinge als vereinbar mit päpstlicher Autorität zu präsentieren, ohne diese Privatmeinung mit dem Vatikan zu koordinieren. Das konnte funktionieren, wie die Karriere von Joseph Ratzinger (1927–2022), als Papst Benedikt XVI amtierend 2005–2013 beweist, aber ebenso war es wahrscheinlich, dass eine zu voluntaristische Einschätzung päpstlicher Gedankengänge verheerende Folgen für die inneren Verhältnisse der katholischen Welt in einem Land haben konnte. So nahm der in den 1950er- und 1960er-Jahren bedeutendste und heute weitgehend in die Vergessenheit gedrängte deutsche Moraltheologe Bernhard Häring (1912–1998) aufgrund der freundlichen Aufnahme neuer Techniken in Anästhesiologie und Psychologie in den 1950er-Jahren an, dass der Vatikan eine hormonelle Empfängnisverhütung ebenfalls als vereinbar mit der Glaubenslehre anerkennen würde [1]. Infolgedessen beriet die Mehrheit der katholischen Beichtväter ihre Pönitenten entsprechend. Nach Erlass der Enzyklika *Humanae Vitae* 1968, die allein eine Verhütung basierend auf Knaus-Ogino gestattete, meldeten sich beispielsweise allein im Raum Frankfurt am Main hunderte von besorgten Priestern bei ihrem Bischof, dass sie – gestützt auf Häring – ihre Gemeindemitglieder über Jahre hinaus falsch beraten hatten und nun nicht wussten, wie sie die Enzyklika erklären sollten [2]. Dies war nicht der erste problematische Bruch innerhalb der katholischen Glaubenslehre im 20. Jahrhundert. Mindestens ebenso verheerend wie *Humanae Vitae *1968 wirkte sich im Jahre 1917 der Erlass des *Codex Iuris Canonici* aus. Bis zu diesem Zeitpunkt galt für die katholische Welt Abtreibung als kein Verbrechen, wenn sie vor dem 40. Tag nach der Befruchtung erfolgt war. Denn ab diesem Zeitpunkt erst sei der Embryo belebt, hatte der Kirchenlehrer Thomas von Aquin (1225–1274) festgelegt. Er und seine Scholastik waren im Jahre 1879 durch Papst Leo XIII (1810–1903, amt. 1878–1903) mittels der Enzyklika *Aeterni Patris* zur allgemein gültigen und unhinterfragbaren Grundlage der katholischen Moraltheologie und Gesellschaftslehre erklärt worden [3]. Dies hinderte den Vatikan nicht daran, einerseits an der Scholastik festzuhalten und jeden Verstoß dagegen drakonisch zu ahnden und sie zugleich in Teilen 1917 außer Kraft zu setzen. Denn ab jetzt galt der Embryo mit dem Tag der Zeugung als beseelt.

Die katholische Kirche befand sich also bereits lange vor dem II. Vatikanischen Konzil in einer schwierigen diskursiven Situation. Sämtliche Vorgaben im medizinisch-ethischen Bereich unterlagen einer Art Fragwürdigkeitsklausel, da ihre Gültigkeit aufgrund des angegriffenen wissenschaftstheoretischen Fundaments nicht unzweifelhaft war. Der Boden für eigenständige Interpretationen durch Autoritäten vor Ort war damit ungewollt bereitet.

## Kirchliche Festlegungen und urologische Fragestellungen

**Figure 1:**
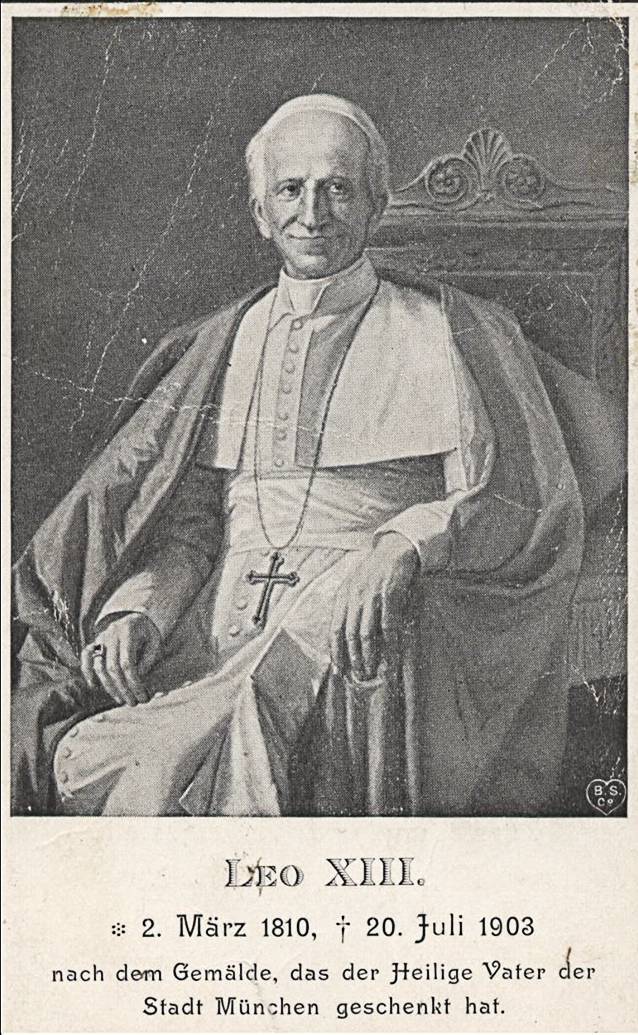

Der Anspruch auf körperliche und seelische Gesundheit wurde erstmals ausdrücklich in der Sozialenzyklika *Rerum Novarum* 1891 festgehalten. Dies betraf jedoch nicht jene Katholiken, die sich mit Prostituierten einließen und sich mit einer Geschlechtskrankheit infizierten. Diese galt als gerechtfertigte Strafe Gottes, wie der Vatikan bereits 1828 in einem Lehrschreiben festhielt, das noch immer (theoretisch) Gültigkeit besitzt [4]. Ärzte, die Patienten mit akuten venerologischen Infektionen oder chronischen Folgeekrankungen behandeln, verstoßen somit aus katholischer Sicht gegen den ausdrücklichen Willen Gottes. Ergänzend verbot Papst Leo XIII 1862 die Verwendung von Kondomen. Ebenfalls vom Zugang zu medizinischer Behandlung auszuschließen seien Personen, die sich einer Impfung unterzögen, wie Papst Leo XII (1760–1829, amt. 1823–1829) im Jahre 1824 bestimmte [5]. Das Zuraten des Arztes zu einer HPV-Impfung (humane Papillomviren) *als Impfung* stellt demnach eine schwere Sünde dar, sowohl für den Arzt als auch die Patientin, wobei eigentlich Urologen in besonderem Maße auch in Unterstützung durch die Fachgesellschaft aufgerufen sind, hier Impfungen zu fördern und auszuführen, was auch die Fachgesellschaft wissenschaftlich korrekt tatkräftig unterstützt ([6, 7]; Abb. 1).

Sexualität dient nach katholischer Auffassung grundsätzlich allein der Fortpflanzung, wie Thomas von Aquin (1225–1275) bestimmt hatte. Jeglicher operativer Eingriff zur Unterbindung der Fortpflanzungsfähigkeit ist daher untersagt, letztgültig festgelegt durch Papst Pius XI (1857–1939, amt. 1922–1939) in der Enzyklika *Casti Connubii *vom Dezember 1930 ([8]; Abb. 1 und 2).

Diese untersagte zugleich die Betätigung in der Freikörperkultur.

**Figure 2:**
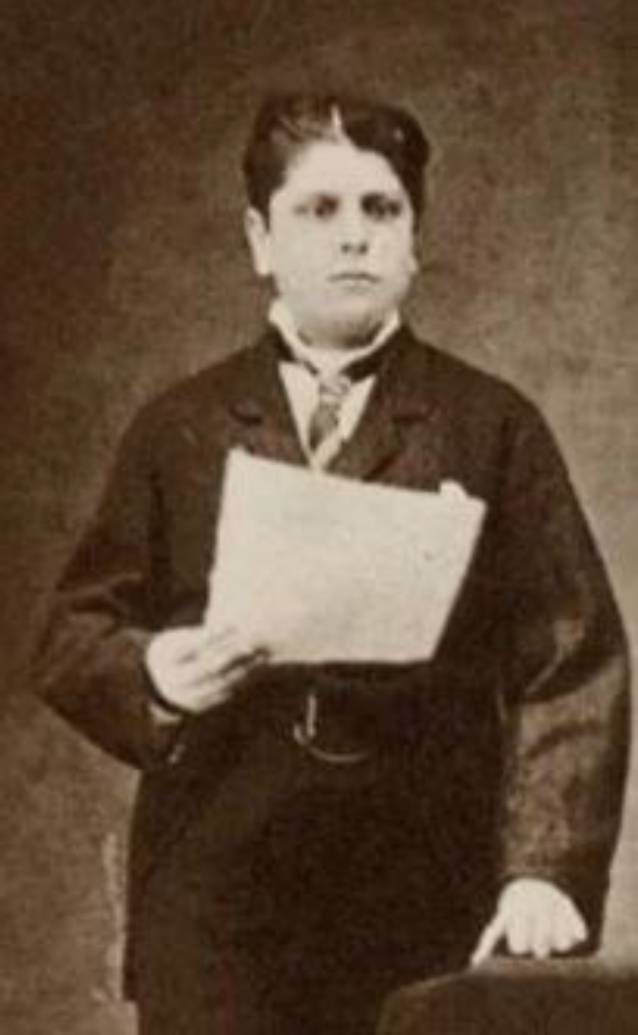

**Figure 3:**
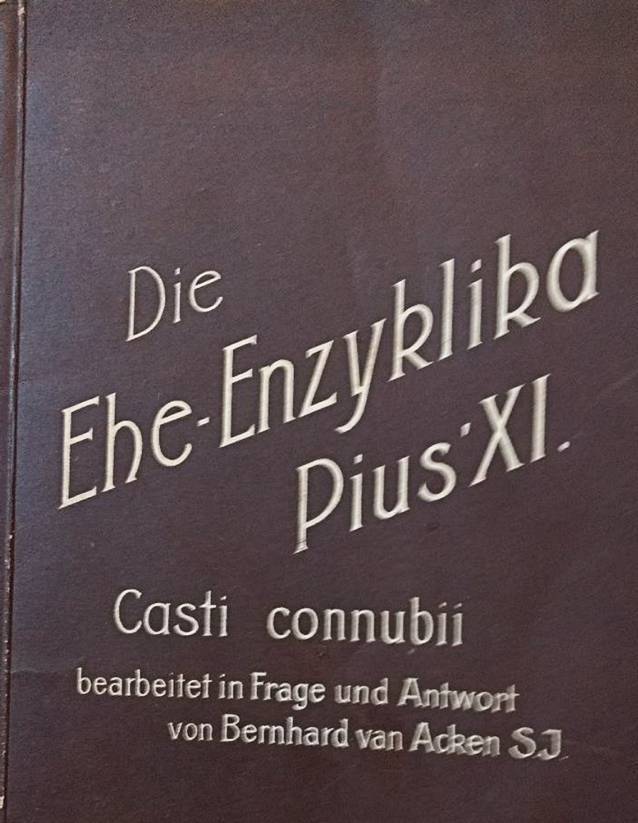

Ein schwieriges Thema stellt für den Vatikan die Kastration dar. Obwohl sie als Eingriff 1587 durch Papst Sixtus V (1521–1590, amt. 1585–1590) als unvereinbar mit der Glaubenslehre bestimmt worden war, sangen noch im 20. Jahrhundert Kastraten in römischen Kirchenchören (Abb. 2; [9]). Personen, denen eine Kastration zugefügt wurde, genießen aus moraltheologischen Gründen den uneingeschränkten Zugang zu medizinischen Leistungen (Abb. 3).

Eine Sterilisation im Rahmen einer medizinischen Indikation gestattete Papst Johannes Paul II (1920–2005, amt. 1978–2005) in Ausnahmefällen im Rahmen des überarbeiteten Katechismus erst ab dem Jahre 1995 (vgl. zu früheren Festlegungen der Kirche [Abb. 3]).

Problematisch ist jedoch die Anwendung von Suggestivtechniken. Diese sind seit 1856 mittels der Enzyklika *Adversus magnetismi abusus *ausdrücklich untersagt [10].

Die sexuell erfüllte Ehe als Heilmittel gegen jede Form von sittlicher Versuchung wurde in *Casti Connubii* betont, aber beispielsweise von Paul VI (1897–1978, amt. 1963–1978) in der Enzyklika *Gaudium et Spes *1965 nicht mehr erwähnt [11]. Abtreibung hingegen ist weiterhin verboten, ebenso jede aktive Form der Sterbehilfe [12].

Schmerzstillende Maßnahmen sind, auch wenn sie lebensverkürzend wirken, durch die Enzyklika *Evangelium vitae *aus dem Jahre 1995 in Einzelfällen, aber nicht generell gerechtfertigt [13].

Als erster Ansprechpartner bei Erkrankungen gilt seit Erlass der Enzyklika *Apostolicae nostrae caritatis* aus dem Jahre 1854 nicht der Arzt, sondern der Priester. Denn der Evangelist Lukas hatte herausgestellt, dass die Kompetenz zur Krankenheilung allein Jesus Christus (Luk 5, 31; Luk 6,19) zustand [14]. Daraus ließ sich ein Prioritätsrecht der Nachfolger Jesu ableiten. Der Priester erlangte seine Erkenntnisse durch die genaue Befragung der Gläubigen im Rahmen der durch den Kirchenlehrer Alphonsus Maria de Liguori (1696–1787) perfektionierten Ohrenbeichte [15]. Priester wurden bis in die 1970er-Jahre im Rahmen ihrer Ausbildung im Bereich der *Pastoralmedizin bzw. Pastoralpsychologie* auch heilkundlich geschult [16].

**Figure 4:**
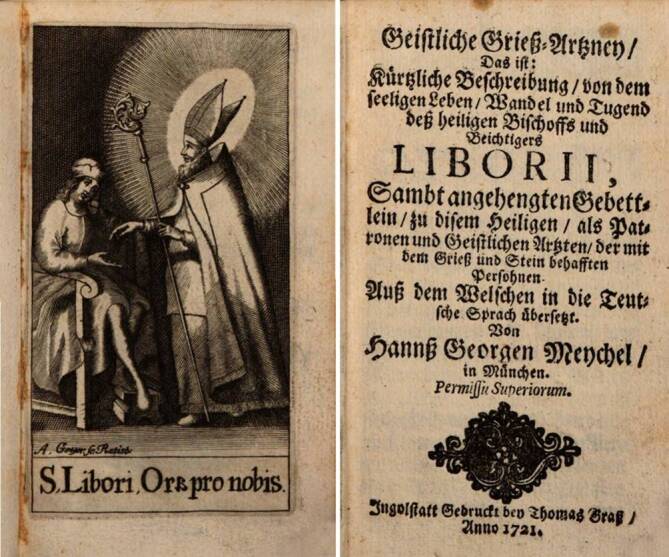

Wenn auch die katholische Kirche niemals lehrte, dass *Heilige* Krankheiten heilen könnten, wurden Heiligen und Heiligenreliquien im Krankheitsfalle eine heilbringende Wirkung zugesprochen. Die Helferqualitäten der Heiligen leiteten sich zwanglos aus deren Biographie und Martyrium ab. Die Anrufung überirdische Mächte bildete somit einen wichtigen Aspekt beim Umgang mit Erkrankungen *Hagiotherapie*. Für den Bereich der Urologie konnten hier besondere Regionale Unterschiede herausgearbeitet werden. Weiterhin spielten die Heiligen als Mittlerfiguren zwischen dem Kranken und Gott eine besondere Rolle ([17]; Abb. 4).

## Priesterliche Medizinalkultur

**Figure 5:**
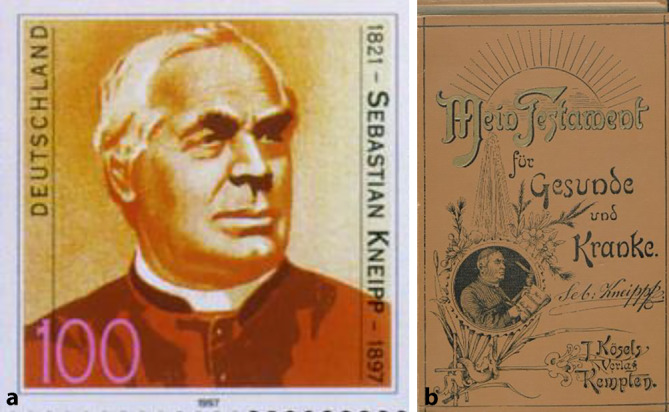

Beeinflusst durch den Erfolg von Sebastian Kneipp (1821–1897) entwickelte sich insbesondere in Süddeutschland und Österreich eine *priesterliche Medizinalkultur*, die noch immer existiert und in ländlichen Gegenden Wirkmächtigkeit besitzt. Kneipp hatte sich vehement gegen das Impfen ausgesprochen und eine naturheilkundliche Diagnose und Therapie empfohlen [18]. Sein bedeutendster Epigone in der zweiten Hälfte des 20. Jahrhunderts war „Kräuterpfarrer“ Hermann Josef Weidinger (1918–2004; [19]; Abb. 5a,b).

## Die Beschneidung Christi – ein Streitpunkt

**Figure 6:**
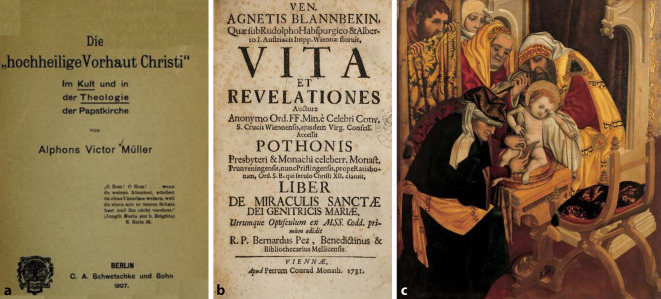

Darüber hinaus gab es einen weiteren Streitpunkt zwischen kirchlichem Lehramt und urologischer Wissenschaftskultur: Urologen führen Beschneidungen durch und besitzen ein hermetisches Wissen über die Beschaffenheit von Vorhäuten und die Techniken der Konservierung. Dadurch bestand aus Sicht der Theologen die Gefahr, dass eine solche Ärztegruppe die in der katholischen Welt verbreiteten und als Reliquien verehrten verschiedenen Teile der angeblichen Vorhaut von Jesus Christus als Fälschung enttarnen könnten. Hierzu gab es seit der Jahrhundertwende kritische Berichte seitens freikirchlicher Agitatoren, die den Reliquienkult kritisierten [20]. Bis zum II. Vatikanischen Konzil blieb die Vorhaut Christi ein fester Bestandteil des katholischen Kultus und durfte nicht hinterfragt werden – am allerwenigsten durch weltliche Ärzte ([21, 22]; Abb. 6a–c).

Den Geistlichen oblag auch die Erziehung der Heranwachsenden in Fragen der Sittlichkeit. Geschlechtliche Aufklärung durch weltliche Autoritäten wie Lehrer oder Ärzte untersagte der Vatikan ausdrücklich bereits 1829 mittels der Enzyklika *Traditi humilitati nostrae* [23].

## Evolutionslehre

Im Laufe des frühen 20. Jahrhunderts hatte die Evolutionslehre allgemeine Verankerung im medizinischen Denken und der Theoriefindung erlangt. Doch katholischen Ärzten war eine entsprechende Orientierung seit der Enzyklika *Quanto conficiamur moeroe *durch Papst Pius IX im Jahre 1863 untersagt. Diese Festlegung wurde durch *Quanta Cura *1864 bestätigt. Das Rundschreiben *Providentissimus deus* von Papst Leo XIII (1882) sprach sich ebenfalls gegen „naturwissenschaftliche Irrtümer“ aus [24]. Doch Charles Darwins (1809–1882) Schlüsselwerk *Origin of Species* landete nie auf dem Index. Die Lektüre war und ist gestattet, die Nutzung der Inhalte zu egal welchem Zweck bleibt verboten.

## Auswege und Hilfen in der Vergangenheit

**Figure 7:**
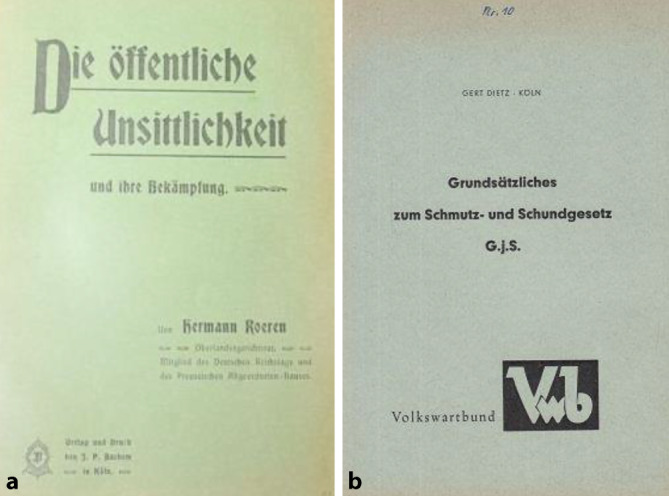

Obwohl der Vatikan im Laufe des 19. Jahrhunderts klare Regelungen für Erkrankungen des menschlichen Unterleibs aufgestellt hatte, zeigte sich insbesondere der deutsche Episkopat im naturwissenschaftlichen Zeitalter als kompromissbereit. Infolge des Kulturkampfes im deutschen Kaiserreich waren geistliche und weltliche katholische Eliten bemüht, die von der protestantisch geprägten preußischen Führungsschicht unterstellte „Inferiorität“ der katholischen Welt zu bekämpfen [25]. Dies beinhaltete insbesondere Anstrengungen zur Rezeption naturwissenschaftlicher und heilkundlicher Erkenntnisse. Nach außen trat die katholische Welt geschlossen auf und von Bistümern materiell und ideell subventionierte Vorfeldorganisation wie der in Köln beheimatete *Volkswartbund* waren bemüht, dem stets befürchteten „Sittenverfall“ entgegenzuwirken ([26]; Abb. 7).

Jedoch beförderten Moraltheologen und kirchennahe Institutionen die konsequente Übernahme neuester medizinischer Erkenntnisse. Der 1897 gegründete und 1916 durch die Deutsche Bischofskonferenz als Dachorganisation legitimierte Caritasverband setzte in seinen Krankenhäusern und Ausbildungseinrichtungen auf die neuesten medizinischen Diagnose- und Behandlungstechniken. Diese beinhalteten Röntgenstrahlen, Psychotherapie, Impfungen und sämtliche chirurgischen Eingriffe zur Rettung von Menschenleben. Zugleich warnten katholische Ärzte in schrillen Tönen vor den gesundheitlichen Folgen unsittlichen Verhaltens, das sich in der masturbatorischen Betätigung manifestiere. Diese galt als Einstiegsdroge und wurde mit verheerenden Konsequenzen assoziiert: Organische Störungen und Veränderungen des Charakters [27], Depressionen [28], Alkoholismus [29], Schwächung von „Gehirn und Rückenmark“ [30], Gedächtnisverlust [31], Hysterie und Lähmungserscheinungen [32] oder Erscheinungen, wie man sie von jahrelangem Alkohol- oder Drogenmissbrauch kenne [33]. Bereits die Lektüre unsittlicher Schriften, wozu ausdrücklich auch Comics gerechnet werden, münde in der geistig-sittlichen Verwahrlosung [34]. In anderen Worten: Die naturwissenschaftlich interessierten oder aus beruflichen Gründen damit assoziierten Teile des katholischen Deutschlands akzeptierten stillschweigend die Hinfälligkeit zahlreicher päpstlicher Verlautbarungen und kompensierten diese laxe Haltung durch eine überzogene Propaganda bei Einzelaspekten des täglichen Lebens, die nicht direkt mit einer ärztlichen Behandlung verbunden waren. Dadurch versicherten sich die katholischen Ärzte und die mit ihnen verbündeten Moraltheologen ihrer unbedingten Loyalität gegenüber Rom und verfügten zugleich über ein wirksames Instrument zur steten Kontrolle der eigenen Gläubigen. Für Urologen bedeutete dies bereits seit der Wende zum 20. Jahrhundert, dass wenn sie nicht gegen fundamentale Vorgaben der Glaubenslehre verstießen, sie ihre modernsten Techniken zur Anwendung bringen durften. Zu nachhaltigen Konflikten führte diese Praxis in den 1930er-Jahren, als gerade Urologen oder Chirurgen die Durchführung der eugenischen Sterilisationen im Nationalsozialismus zufallen sollte. Diese Eingriffe stellten einen direkten Verstoß gegen die Enzyklika *Casti Connubii* und somit eine schwere Sünde dar. Das schloss beteiligte katholischen Urologen oder Chirurgen an Sterilisationsoperationen automatisch aus der Kirche aus und verwehrte ihnen die Erlangung des Seelenheils (ewige Verdammnis).

## Zusammenfassung – Ausblick auf die Zukunft

Die katholische Moraltheologie hat seit dem Amtsantritt von Papst Franziskus I (geb. 1936, amt. seit 2013) eine Reihe von Modifikationen vorgenommen, die auf eine Bereitschaft zur Akzeptanz einer Situationsethik hindeuten. Dies sind jedoch Entscheidungen eines einzelnen Papstes, nicht der Kirche als solche, wie dies im Falle der Enzyklika *Humanae Vitae *der Fall war, die als Ergebnis eines Konzils angesehen werden kann.

Es ist also durchaus möglich, dass der nächste Papst hier wieder Korrekturen vornehmen wird. Jedoch lässt sich eine grundsätzliche Bereitschaft erkennen, einmal eingeschlagene Pfade und Entscheidungsfindungen zwar bestehen zu lassen, aber in gewisser Weise einen „linguistic turn“ zu vollziehen. Bestimmte Handlungen werden als solche anders bezeichnet, wodurch die zuvor erlassenen Verbote keine Berücksichtigung mehr finden müssen. Eventuell ist es so möglich, gewisse Tätigkeiten, Lebensstile oder sexuelle Orientierungen mit neuen Definitionen zu versehen, wie dies amerikanische katholische Ethiker 2013 vorschlugen. Würde man beispielsweise „Masturbation“ nicht mehr „Masturbation“ sondern „Greifen an den Genitalien“ nennen, so stünde hierzu in keiner Enzyklika eine Ablehnung und eine Neuform(ul)ierung der kirchlichen Haltung wäre möglich [35].

Auf die urologische Praxis übertragen, würde das bedeuten, dass wenn man die Folgen einer Infektion mit Geschlechtskrankheiten nicht mehr ausdrücklich als solche bezeichnet, sondern die daraus erwachsenen gesundheitlichen Probleme separat benennt, es keinen Widerspruch mit der katholischen Glaubenslehre darstelle, geschlechtskranke Patienten zielorientiert zu therapieren. Auch eine HPV-Impfung lässt sich als glaubenskonformer Eingriff bezeichnen, wenn man sie als Teil einer sittlichen Ermahnung zur Meisterung der Herausforderungen einer weitgehend entkirchlichten Welt präsentiert.

Gerade im Kontext der nicht enden wollenden Missbrauchsdebatten wäre eine Neudefinition, welche Formen sexuellen Begehrens glaubenskonform sind und welche nicht, dringend erforderlich. Der Vatikan versucht sich seit einigen Jahren als Teil des „aufgeklärten Westens“ und somit als Gegenpol zur islamistischen Terrorgefahr zu positionieren [36]. Damit stellt sich die katholische Kirche (ungewollt?) in die Tradition der säkularen Welt und unterwirft sich praktisch deren Wertvorstellungen. Dies kann die Haltung des Vatikans zu gesundheitlichen, medizinethischen und (sexual-)politischen Entwicklungen und Festlegungen nicht unberührt belassen.

Es liegt natürlich an den jeweiligen Entscheidungsträgern vor Ort, ob sie einer solchen Interpretation folgen, also der deutschen Bischofskonferenz und den Beichtvätern in den Gemeinden sowie den Krankenhausseelsorgern. Hier bedarf es eines guten Verhältnisses zwischen Arzt und Priester, das jedoch heutzutage häufig fehlt. Denn Urologen an sich sind schon eine sehr seltene Spezies, katholische Urologen, die ihren Glauben ernst nehmen, dürften zu einer besonders marginalen Gruppe von Ärzten gehören. Eventuell wäre es sinnvoll, zum Wohle der Patienten, hier mit entsprechend interessierten Kollegen in Austausch zu treten. Man muss kein gläubiger Katholik sein, um gläubigen Katholiken ärztlich beizustehen. Wenn es gelingt, in die Denkwelten muslimischer Patienten einzutauchen, dann sollte dies bei Katholiken auch kein Problem darstellen. Eventuell kann die von Papst Franziskus empfohlene verstärkte Einbindung von Laien hier Abhilfe schaffen, indem die katholische Welt das in den 1970er-Jahren entwickelte protestantische Konzept der *Clinical Pastoral Education* bzw. des *spiritual care* für sich entdeckt [37, 38].
